# Morphological Retrospective Study of Peritoneal Biopsies from Patients with Encapsulating Peritoneal Sclerosis: Underestimated Role of Adipocytes as New Fibroblasts Lineage?

**DOI:** 10.1155/2015/987415

**Published:** 2015-08-19

**Authors:** Monika Tooulou, Pieter Demetter, Anwar Hamade, Caroline Keyzer, Joëlle L. Nortier, Agnieszka A. Pozdzik

**Affiliations:** ^1^Laboratory of Experimental Nephrology, Department of Biochemistry, Faculty of Medicine, Université Libre de Bruxelles (ULB), 1070 Brussels, Belgium; ^2^Department of Pathology, Cliniques Universitaires de Bruxelles (CUB), Erasme Hospital, Université Libre de Bruxelles (ULB), 1070 Brussels, Belgium; ^3^Department of Nephrology, Cliniques Universitaires de Bruxelles (CUB), Erasme Hospital, Université Libre de Bruxelles (ULB), 1070 Brussels, Belgium; ^4^Department of Radiology, Cliniques Universitaires de Bruxelles (CUB), Erasme Hospital, Université Libre de Bruxelles (ULB), 1070 Brussels, Belgium

## Abstract

*Background*. Encapsulating peritoneal sclerosis (EPS) is a rare but serious complication of peritoneal dialysis (PD). Besides the endothelial-to-mesenchymal transition (EMT), recently peritoneal adipocytes emerged as a potential source of fibrosis. We performed immunohistochemistry to approach EMT and to localize peritoneal adipocytes in peritoneal biopsies from PD-related EPS patients. *Material and Methods*. We investigated tissue expression of podoplanin, cytokeratin AE1/AE3 (mesothelium), calretinin (adipocytes), alpha-smooth muscle actin [*α*-SMA] (mesenchymal cells), interstitial mononuclear cell inflammation, and neoangiogenesis (CD3, CD4, CD8, CD20, CD68, and CD31 immunostainings, resp.). *Results*. Three patients (1 man/2 women; 17, 64, and 39 years old, resp.) developed EPS after 21, 90, and 164 months of PD therapy. In patients with EPS, we observed (1) loss of AE1/AE3 cytokeratin+ mesothelial cells without any evidence of migration into the interstitium, (2) disappearance of adipose tissue, (3) diffuse infiltration of calretinin+ cells in the areas of submesothelial fibrosis with a huge number of *α*-SMA and calretinin+ fusiform cells, and (4) increased vascular density. *Conclusion*. We report that the involvement of EMT in peritoneal fibrosis is difficult to demonstrate and that the calretinin+ adipocytes might be an underestimated component and a new source of myofibroblasts in peritoneal remodeling during PD-related EPS.

## 1. Introduction

Peritoneal dialysis (PD) is a first choice and successful home-based dialysis modality for patients with end-stage kidney disease (ESKD), with great advantages for their quality of life: preservation of residual renal function, no vascular access requirement, and possibility of continuing scholar or professional activities [1, 2]. Moreover, the International Society of Nephrology and International Society of Peritoneal Dialysis strongly advise PD therapy for acute kidney injury, especially in a pediatric population [3]. Despite the abovementioned benefits, PD therapy deserves some particular attention. Indeed, long-term PD may prompt the remodeling of peritoneal membrane and loss of mesothelial cells monolayer, increase in vascular density with diabetes-like vascular abnormalities (typical PD-associated venular subendothelial hyalinosis), vascular calcifications, and interstitial fibrosis [4]. These pathological structural changes of the peritoneal membrane are most frequently followed by functional consequences resulting in progressive loss of peritoneal membrane ultrafiltration capacity leading to discontinuation of PD therapy [5, 6].

Encapsulating peritoneal sclerosis (EPS) is still worrying and is an uncommon life-threatening complication of peritoneal dialysis with an incidence of 0.5 to 2.5% and a high mortality rate (25% to 55%) [7]. Following the International Society of Peritoneal Dialysis guidelines, diagnosis criteria of EPS include the association of clinical symptoms, radiological and histological findings [8]. The main pathological feature of EPS consists in a marked peritoneal fibrosis; however it lacks specificity as various degrees of submesothelial thickening have been reported in patients with chronic kidney disease [9, 10]. The pathophysiology of EPS is still unknown [11]. Nowadays, it appears that peritoneal fibrosis cannot be entirely explained on the basis of the simple model of wound healing (a three-phase model including injury of mesothelial cells, inflammation, and repair) [7, 12, 13]. This epithelial to mesenchymal transdifferentiation (EMT) process, first described in kidney fibrosis, states that the mesothelial cells change into a mesenchymal phenotype, migrate to submesothelial areas, and differentiate into “activated myofibroblasts,” the cells responsible for fibrosis. The EMT was proposed to be involved mainly in the early stage of peritoneal fibrosis [14]. Recent data demonstrate that the resident peritoneal interstitial cells are early activated following the aggression of mesothelial cells (or epithelium), the inflammatory infiltrate, and profibrosing cytokine microenvironment. Currently, new data suggest that peritoneal adipocytes could also contribute to this pathological process [15].

In our study, we approached the EMT process and adipocytes involvement in peritoneal fibrosis in a morphohistological retrospective analysis of 6 peritoneal tissue biopsies (3 cases with PD-related EPS, 2 cases with normal peritoneal tissue, and 1 case with acute peritonitis, for a histological study during an acute inflammatory process). Our presented data show that the resident peritoneal adipocytes represent an underestimated source of peritoneal myofibroblasts in PD-induced EPS.

## 2. Material and Methods

The study was evaluated and approved by the Local Ethic Committee (Erasme Hospital number P2014/184). We included peritoneal biopsies samples of the 3 patients with EPS diagnosed between 1995 and 2013 in our center. We selected the 2 control patients with normal peritoneum (randomly selected in our database of patients who had abdominal surgery in our center and with normal renal function). We also analyzed 1 case with acute peritonitis, with normal renal function (so without any ESKD or peritoneal fibrosis), in order to evaluate the hypothesis of early crosstalk between inflammatory cells, mesothelial cells, and adipocytes. For EPS diagnosis, we applied the clinical and biological criteria adapted from Nakamoto [16]: (i) stage 1 (pre-EPS), characterized by loss of ultrafiltration, high transport status, hypoproteinaemia, bloody dialysate, ascites, and peritoneal calcifications; (ii) stage 2 (inflammatory), increase in C-reactive protein level and white blood cell count, fever, weight, and appetite loss; (iii) stage 3 (encapsulating or progressive), disappearance of signs of inflammation and appearance of signs of ileus (nausea, vomiting, abdominal pain, and constipation), abdominal mass with ascites, and (iv) stage 4 (obstructive or cocooning). We used the formalin-fixed peritoneal tissue embedded in paraffin blocks available in the files of the pathology department of our hospital. Medical records analysis of included cases provided epidemiological data (age, gender), PD characteristics (dialysis modality, type of solution, and PD duration), clinical, radiological, and laboratory parameters at EPS diagnosis, time between renal transplantation and the onset of EPS symptoms, and prescribed immunosuppressive agents. Treatment modalities and outcomes of EPS were also recorded and included.

### 2.1. Standard Stainings and Immunohistochemistry

Standard stainings (Masson's trichrome and haematoxylin-eosin (HE)) were used to illustrate peritoneal fibrosis, mesothelial cells, and inflammatory infiltrate. The entire sample of each peritoneal tissue specimen was analyzed by optical microscopy using low (×40 and ×100), medium (×200), and high (×400) magnifications (Carl Zeiss, Oberkochen, Germany).

The study of tissue expression of podoplanin, AE1/AE3 cytokeratin (mesothelial phenotype), calretinin (expressed by mesothelial cells and adipocytes), vimentin (mesenchymal phenotype), *α*-SMA (myofibroblasts), CD4, CD8, CD20, CD68 (immunophenotyping of inflammatory cells), and CD31 (endothelial cells marker) was performed on sections of 4 *μ*m thickness using an immunohistochemistry analysis technique (Ventana XT-Discovery, Tucson, USA). Immunoperoxidase procedures counterstained with hematoxylin were applied. We chose the following human tissues as positive controls for immunohistochemistry of used antibodies: tonsil for immunostaining of anti-CD4, anti-CD8, anti-CD20, and anti-CD68 antibodies (3-membrane and 1-membrane-cytoplasm patterns, resp.); colon for anti-AE1/AE3 cytokeratin antibodies (cytoplasm pattern); vessels for anti-*α*-SMA and anti-CD31 antibodies (cytoplasm pattern); sarcoma for anti-vimentin antibodies (cytoplasm pattern); and adipose tissue for anti-calretinin antibodies (nuclear and cytoplasm pattern). The negative controls were performed in the absence of primary antibodies and showed no staining (Table 1).

### 2.2. Quantification of Immunostainings

The semiquantitative score for cytokeratin AE1/AE3, calretinin, vimentin, and *α*-SMA expression was applied as follows: strong expression (+++), moderate expression (++), low expression (+), or no expression (0).

The quantitative analysis of CD4 expression (subpopulation of T helper cells and infiltration macrophages), CD8 expression (subpopulation of cytotoxic/suppressor T cells), CD20 (B cells population), and CD68 (circulating monocyte and macrophages) was performed by calculating the number of positive cells per field (high magnification, ×400).

For quantitative analysis of CD31 expression (vascular density), we counted CD31 positive vessels per field (medium magnification, ×200).

We analyzed 20 fields at random in each case using an optical microscope (Carl Zeiss, Oberkochen, Germany).

## 3. Results 

### 3.1. Clinical, Biological, and Radiological Characteristics of Studied Patients

The clinical and biological characteristics at diagnosis of EPS are summarized in Table 2. We identified a 17-year-old man (case  1) and two women aged 64 years (case  2) and 39 years (case  3) treated with PD (for 21, 90, and 164 months, resp.), who developed EPS after shift to hemodialysis (case  1) and after first (case  2) and second kidney transplantation (case  3).

**Figure 1:**
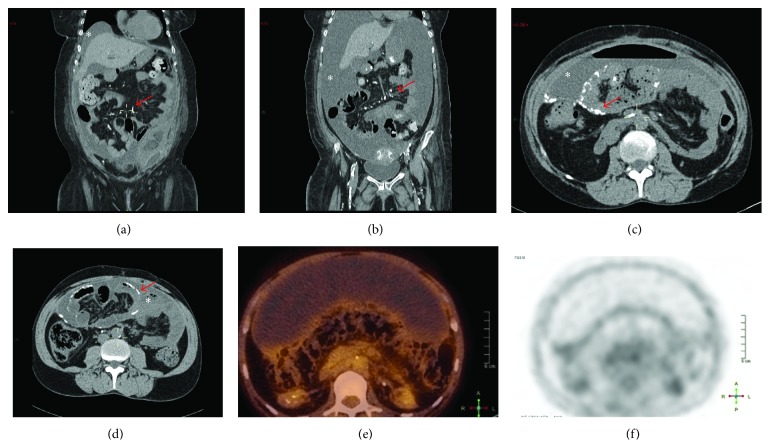
Radiological findings suggesting encapsulating peritoneal sclerosis (EPS). (a, c and b, d correspond to cases EPS 2 and EPS 3, resp.) Abdominal computed tomography without contrast agent showed massive nonloculated fluid and bowel loops drowned into the center of the abdominal cavity suggestive of bowel adhesions. As compared with EPS case  2, EPS case  3 presented more abundant abdominal ascites, marked narrowing of bowel lumen and thickening of visceral and parietal peritoneum, absence of dilated bowel loop, air-fluid levels or entrapped fluid collections, and several calcifications in both parietal and visceral peritoneum. (e and f, case EPS 3) Fluorodeoxyglucose^18^ positron emission tomography (F^18^ PET) scan showed a mild increase in the tracer uptake in the peritoneal areas.

In all cases, the diagnosis was suspected because of digestive symptoms, systemic inflammation, and normocytic anemia. Furthermore, cases 2 and 3 had hemorrhagic ascites. Their abdominal CT scan showed, in addition to abundant ascites, diffuse peritoneal calcifications, with parietal and visceral involvement (Figure 1). Positron emission tomography with [^18^F] fluorodeoxyglucose (FDG-PET) was available only in one case (case  3) and demonstrated significant FDG uptake by the parietal peritoneal membrane (Figure 1).

### 3.2. Histological and Immunohistochemical Findings

**Figure 2:**
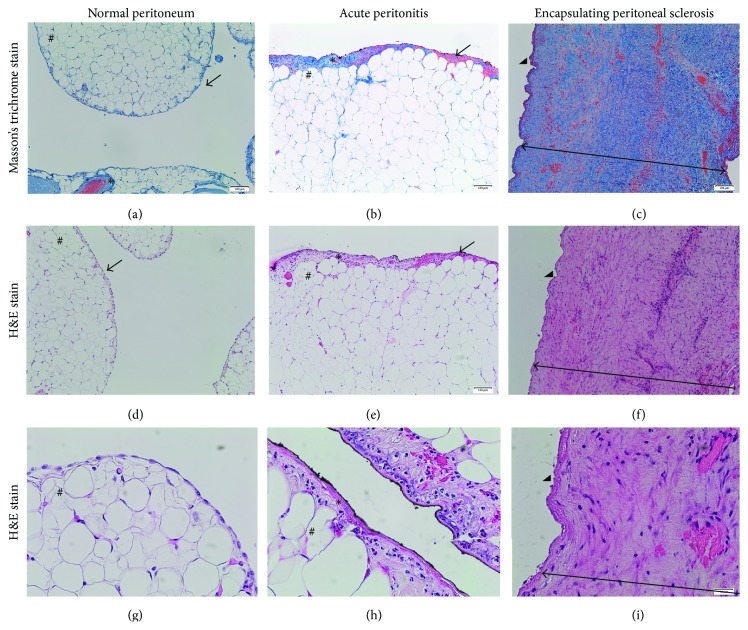
Peritoneal biopsies representative photomicrographs of haematoxylin-eosin and Masson's trichrome stainings in studied cases (a, d, and g). Mesothelium (→) and adipocytes (#) in the submesothelial area in normal peritoneal membrane (b, e, and h). Well preserved mesothelium with hyperplasic mesothelial cells and increase in conjunctive tissue (*∗*) associated with interstitial submesothelial infiltrate mainly polymorphonuclear neutrophils (++) in acute peritonitis case.* Superficial black lining related to the surgery technique (use of Indian ink).* (c, f, and i) Disappearance of mesothelium (arrowhead), major submesothelial fibrosis containing mainly mononuclear cells, and sever fibrosis (*←*→) in case of EPS. Original magnifications: (a–f) ×10, (g–i) ×40.

Compared with the normal peritoneal tissue biopsies (controls), massive submesothelial thickening corresponding to fibrosis was associated with the disappearance of the mesothelium in all EPS cases (Figure 2). In controls, we observed a distinct monolayer of cubic mesothelial cells closely attached to one another and affixed to the thin basement membrane, which was in direct contact with a waste area of adipose tissue. In the control 3 biopsy (acute peritonitis), mesothelium was well preserved but we found a marked hyperplasia of mesothelial cells attached to the basement membrane and the apposition of inflammatory connective tissue containing predominantly polymorphonuclear neutrophils infiltrate (Figure 2).

### 3.3. The Mesothelial Cells in Controls, Acute Peritonitis, and EPS Biopsies

**Figure 3:**
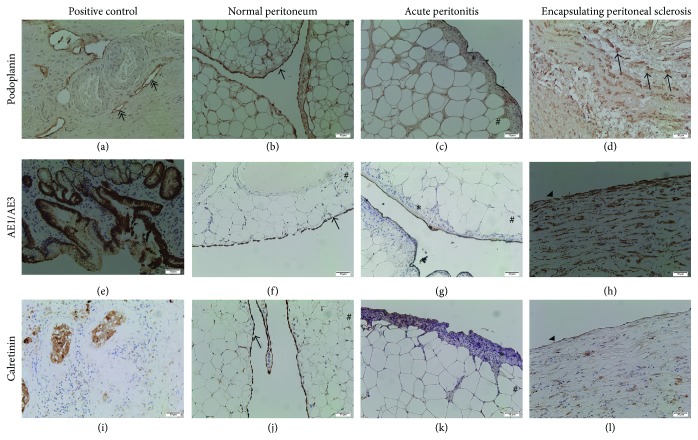
Peritoneal biopsy representative photomicrographs of mesothelial phenotype markers expression: podoplanin (a–d), cytokeratin AE1/AE3 (e–h), and calretinin (i–l). Positive control of immunostainings for used antibodies (internal controls) (a, e, and i), control case: normal peritoneum (b, f, and j), case of acute peritonitis (control 3, c, g, and k), and case  1 of encapsulating peritoneal sclerosis (EPS) (d, h, and l). Physiologic expression of podoplanin (cytoplasm of endothelium in lymphatics (*↠*)), AE1/AE3 cytoplasm in epithelial cells of stomach and calretinin (cytoplasm in epithelial cells) (a, e, and i, resp.). (b, f, and j) Intact peritoneal membrane biopsy in controls; mesothelial cells (→) with obvious expression of adipocytes (#). (c, g, and k) Acute peritonitis case: hyperplasia of mesothelial cells.* Superficial black lining related to the surgery technique (use of Indian ink).* (d, h, and l) EPS case: loss of mesothelial cells (arrowheads) as attested by absence of podoplanin, AE1/AE3, and calretinin expressions. Note hyperplasia of mesothelial cells expressed all mesothelial markers podoplanin, cytokeratin AE1/AE3, and calretinin located in interstitial areas. The architecture of interstitium is strongly modified and contains several calretinin+ fusiform (fibroblasts-like) cells (arrow). Immunoperoxidase staining counterstained with haematoxylin. Original magnification: (a–l) ×20.

In all 3 control peritoneal biopsies, the thin layer of mesothelial cells strongly expressed podoplanin, cytokeratin AE1/AE3, and calretinin (mesothelial markers) (Figure 3, Table 3); these data are consistent with literature [17].

Podoplanin expression was also found in the endothelial cells of lymphatic vessels and mesothelium (Figures 3(a)–3(d)). In case with acute peritonitis, mesothelial cells did not express podoplanin. Only in case  1 of EPS, hyperplastic podoplanin positive mesothelial cells were clearly identified in the interstitium, which had a completely remodeled architecture. In this case, an acute inflammatory component was present at the time of peritoneal biopsy. Interestingly, in case  3 of EPS, we objectified increased podoplanin expression by deep vascular structures. This may suggest an increase in lymphatic vessels density.

High expression of cytokeratin AE1/AE3 by mesothelial cells was observed in the 3 controls but was lacking in all cases of EPS (Figures 3(e)–3(h), Table 3). In the acute peritonitis case, mesothelial cells did not express cytokeratin AE1/AE3. Despite basement membrane rupture, we did not find AE1/AE3+ cells migrating to interstitial areas. Only in case  1 of EPS, hyperplastic cytokeratin AE1/AE3+ mesothelial cells were clearly identified in the remodeled interstitium. These cells did not present typical for myofibroblasts fusiform morphology.

Expression of calretinin in mesothelium was similar to AE1/AE3. Interestingly, expression of calretinin by adipocytes, although in low intensity (+), was constant in all of our 3 controls and acute peritonitis. Nevertheless, we clearly objectified an interstitial accumulation of calretinin positive spindle cells in the 3 EPS cases (Figures 3(i)–3(l), Table 3).

### 3.4. Interstitial Infiltration by Polymorphonuclear Cells in Acute Peritonitis and by Mononuclear Cells in EPS Biopsies

**Figure 4:**
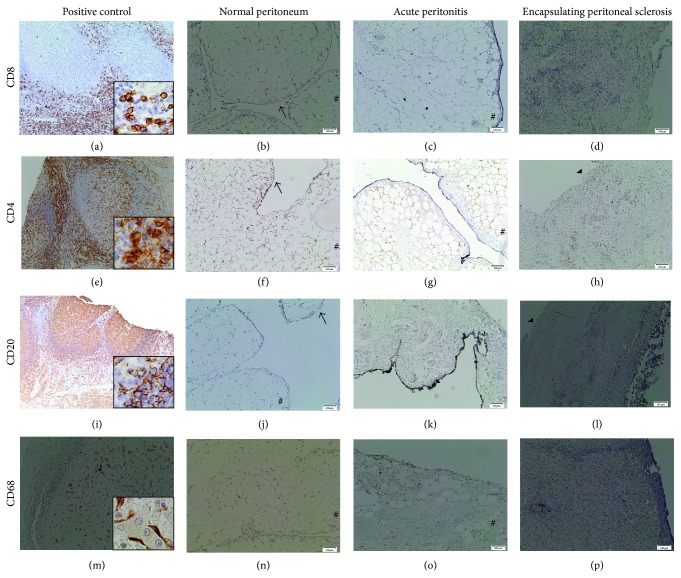
Representative photomicrographs demonstrating histopathological data of interstitial inflammatory cells infiltration in parietal peritoneal tissues biopsy using immunostaining of CD8 (a–d), CD4 (e–h), CD20 (i–l), and CD68 (m–p). Positive control of immunostainings for used antibodies (internal controls) (a, e, i, and m), control: normal peritoneum (b, f, j, and n), case of acute peritonitis (control 3; c, g, k, and o), and case  1 of encapsulating peritoneal sclerosis (EPS) (d, h, l, and p). Absence of mononuclear cells in normal peritoneum. Weak expression of all CD8, CD4, CD20, and CD68 in conjunctive tissue areas adjacent to mesothelium in case with acute peritonitis characterized mainly by polymorphonuclear cells infiltration. Marked interstitial inflammatory cells identified (d) several CD8+ cells (T lymphocytes), (h) few CD4+ cells (T lymphocytes), and (l) CD20+ (B cells), which were accompanied by several (f) CD68+ cells (macrophages) diffusely infiltrating fibrotic areas. Immunoperoxidase staining counterstained with hematoxylin. Original magnification: (a–p) ×20. Small pictures (a, e, i, and m): ×40.

In comparison with controls, we found diffuse mononuclear cells infiltration containing macrophages, T cells, and few B cells in all EPS cases. We observed a marked heterogeneity between the EPS cases, with a highly variable degree of mononuclear cell infiltration, containing mainly CD68+ and CD8+ cells. Those cells were absent in the interstitium from patient with acute peritonitis that contained several polymorphonuclear cells (Figure 4, Table 3).

### 3.5. The Mesenchymal Cells in Controls, Acute Peritonitis, and EPS Biopsies

**Figure 5:**
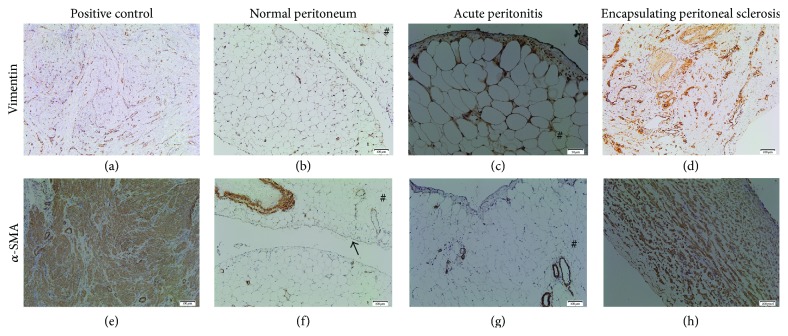
Representative photomicrographs demonstrating histopathological data of mesenchymal markers expression in parietal peritoneal tissues biopsy using immunostaining of vimentin (a–d) and alpha-smooth muscle actin (*α*-SMA) (e–h). Positive control of immunostainings for used antibodies (internal controls) (a, e), control: normal peritoneum (b, f), case of acute peritonitis (control 3; c and g), and case  1 of encapsulating peritoneal sclerosis (EPS) (d and h). (a, e) Normal peritoneum, expression of vimentin (→) limited to few interstitial cells and of *α*-SMA expression to vascular walls. (c, g) Acute peritonitis: interstitial vimentin+ cells, lack of expression of vimentin in mesothelial cell layer; *α*-SMA found only in the vessels. (d, h) Case of encapsulating peritoneal sclerosis (EPS): absence of expression of both markers in the mesothelial cell layer. Note diffuse accumulation of vimentin+ cells identifying interstitial mesenchymal cells and *α*-SMA immunostaining of numerous interstitial cells entrapped in the fibrotic areas reflecting the presence of myofibroblasts. Immunoperoxidase staining counterstained with haematoxylin. Original magnification: (a–g) ×20. (h) ×4.

**Figure 6:**
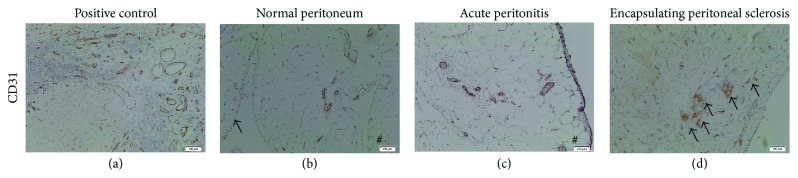
Representative photomicrographs demonstrating histopathological data of endothelial cells marker expression in parietal peritoneal tissues biopsy using immunostaining of CD31 (a). Positive control of immunostainings for used antibodies (internal controls) (a), control: normal peritoneum (b), case of acute peritonitis (control 3; c), and case  1 of encapsulating peritoneal sclerosis (EPS) (d). As compared with normal and acute peritonitis cases, increases in endothelial CD31 expression identifying nonlymphatic vascular network were localized in the deep areas of peritoneum and reflected increased vessels density. Immunoperoxidase staining counterstained with hematoxylin. Original magnification: (a–d) ×20.

The constitutional expression of *α*-SMA was mainly found in the vessels and in some rare interstitial cells. In all cases, many spindle cells expressed vimentin and *α*-SMA and corresponded to mesenchymal cells and myofibroblasts accumulation (Figure 5). A significant increase in vascular density (CD31 positive endothelial cells) was observed in all EPS cases, as compared with controls (Figure 6, Table 3).

## 4. Discussion

The main finding of our study was the discovery of fusiform calretinin positive cells in areas of severe submesothelial fibrosis in all EPS patients. In normal peritoneal tissue biopsy, weak calretinin staining was found in adipocytes and more enhanced in mesothelial cells according to literature [18]. Calretinin (29 kDa calbindin) is a vitamin D-dependent calcium-binding protein coded by the* CALB2* gene and emerges as a multifunctional protein associated with cells development, proliferation, differentiation, and cell death [19].

Our EPS cases were recognized after discontinuation of PD, suggesting the need for vigilance of the nephrologists taking care about transplanted or on hemodialysis patients previously treated by PD [7]. As the incidence of EPS increases with time after renal transplantation [11], the immunosuppressive regimen involvement in the pathogenesis of posttransplant EPS remains an unresolved question.

Several other risk factors have been identified such as high glucose concentration dialysate, long duration of PD, young age, and the use of beta-blockers or cyclosporine and peritonitis [7, 11, 16]. One constant in these factors is the duration of PD, confirmed recently by two independent groups [20, 21]. Indeed, more severe fibrosis is observed in transplanted patient with longest PD vintage and who had a high glucose exposure.

Besides the duration of PD therapy, the number of peritonitis episodes is still a significant risk factor [20, 21]. For this reason, in our morphohistological study we included a case of acute peritonitis not related to PD or ESKD, in order to evaluate the hypothesis of early crosstalk between inflammatory cells and resident peritoneal membrane cells (adipocytes and mesothelial cells). These interactions could be an early link between the PD-related peritonitis (acute inflammation) and peritoneal fibrosis [22]. Interestingly, we found some morphological similarities between mesothelial cells hyperplasia observed in acute peritonitis and in EPS cases, which is in accordance with the findings by others [17]. The reason for mesothelial cells hyperplasia is unknown; however it may be postulated that the cells are activated secondarily by cytokines (released by inflammatory cells) or by hypertonic solutions.

Peritoneal thickening and lowering in the lumen/vessels diameter ratio related to uremia have been reported in patients with chronic kidney diseases [10]. The EMT process has been proposed as a chief pathway of peritoneal fibrosis, mainly in its early stage [23], so early before beginning PD [10]. Despite extensive studies on EMT [14, 23, 24], many aspects of peritoneal membrane remodeling and EPS remain poorly understood [25, 26] suggesting that additional novel mechanisms and pathways need to be explored [13]. Interpretation of our data is unfortunately limited by the small number of studied biopsies as well as the fact that they were obtained at advanced stages of EPS. Indeed, agreeing to Nakamoto [16], cases  1, 2, and 3 corresponded to pre-EPS and inflammatory and progressive or encapsulating EPS, respectively. Moreover, we observed a highly variable degree of interstitial inflammation. Similar to previous report [17], we did not find a transmembrane migration of mesothelial cells into the interstitium, a pivotal phase of EMT. Our results did not confirm that peritoneal interstitial fibroblasts derive from EMT.

The role of EMT in peritoneal fibrosis has been adapted from mechanisms reported in kidney fibrosis (KF). However today, this model begins to be questioned [27–30]. In fact, resident cells (fibroblasts) are considered as the main source as only 5% of myofibroblasts derived from EMT in experimental models of KF [31]. As in the renal fibrosis, already present resident peritoneal cells should be considered as a potential source for myofibroblasts generation. Indeed, peritoneal adipocytes are pluripotent cells and they are active players in fibrosis [32–34]. Therefore, peritoneal calretinin positive adipocytes might be a new and actually underestimated source of myofibroblasts. As compared with controls, the submesothelial adipose tissue containing several calretinin positive adipocytes completely disappeared; nonetheless numerous fusiform calretinin positive cells were observed in the areas of peritoneal fibrosis in EPS biopsies. Intriguingly, in case of acute peritonitis, we found that a submesothelial layer of inflammatory cells closely bordered adipocytes. Unfortunately because of insufficient quantity of peritoneal tissue biopsies, the expression of adipose cells mRNA was not performed. Besides dialysate, several cytokines and growth factors such as transforming growth factor-beta (TGF-*β*) a pivotal profibrotic cytokine secreted by injured mesothelial cells and/or inflammatory cells could be involved in adipocytes differentiation into the peritoneal fibroblasts [35]. In fact, strong evidence suggests a possible crosstalk between the PD solutions, adipose tissue, and peritoneal fibrosis [15, 32]. Moreover, adipocytes mediate numerous physiological processes, secreted adipokines (leptin, adiponectin), cytokines (TNF*α*, IL-6), and growth factors including transforming growth factor-beta (TGF-*β*) [32]. Leptin stimulates lipolysis and inhibits lipogenesis [32]. In human peritoneal mesothelial cells, it has been reported that glucose increased the leptin mRNA expression and its synthesis. Concomitantly, the leptin receptor was upregulated in mesothelial cells and leptin induced the release of TGF-*β* by mesothelial cells. Interestingly, glucose markedly amplified this process [36]. It must be taken into account that adipocytes could be potentially in direct contact with the glucose contained in PD solution after disruption of mesothelium integrity. Indeed shedding of mesothelial cells into the peritoneal cavity by alteration of cell junctions and basement membrane denudation are induced by recurrent mechanical stress related to the daily variations in intra-abdominal pressure and to turbulences of in- and outflow of PD solutions (volume, number of cycles) [1, 7]. Above data could be a plausible way to explain the observed loss of adipose tissue as our cases were exposed to high glucose concentration PD solutions during a long time.

In conclusion, we report that the involvement of EMT in peritoneal fibrosis is difficult to demonstrate. The calretinin positive cells accumulate in the submesothelial fibrosis so that adipocytes might be an underestimated component and a new source of myofibroblasts in peritoneal remodeling during EPS related to PD.

## Figures and Tables

**Table 1:** Details of immunohistochemistry studies performed on peritoneal tissue biopsies (primary antibodies and corresponding cells specificity, retrieval processes, dilution, and type of secondary antibodies).

Antigen	Specificity	Retrieval (time in minutes)	Dilution	Manufacturer
Secondary antibody: monoclonal				
Podoplanin	Mesothelium, lymphatics endothelium	H1 (20)	1/50	Covance, Princeton, New Jersey, USA

Secondary antibody: polyclonal				
AE1/AE3	Mesothelium	H1 (20)	1/800	Dako, Glostrup, Denmark
Calretinin	Mesothelium	H1 (20)	1/150	Menarini, Zaventem, Belgium
CD8	Cytotoxic/suppressor T cells subpopulation	H1 (20)	1/800	Dako, Glostrup, Denmark
CD20	B cells	H1 (30)	1/3000	Dako, Glostrup, Denmark
CD68	Monocytes/macrophages	H1 (30)	1/100	Menarini, Zaventem, Belgium

Secondary antibody: peroxidase				
CD4	Helper T cells subpopulation	H2 (20)	1/50	Klinipath, Duiven, Netherlands
CD31	Endothelium	H2 (30)	1/1000	Menarini, Zaventem, Belgium
Vimentin	Mesenchymal cells	/	1/100	Klinipath, Duiven, Netherlands
*α*-SMA	Myofibroblasts	/	1/100	Menarini, Zaventem, Belgium

H1: citrate buffer, pH 6.0; H2: EDTA buffer, pH 8.0.

**Table 2:** Patients with encapsulating peritoneal sclerosis: epidemiological characteristics, diagnostic criteria, risk factors, treatment, and outcomes.

	Case EPS 1	Case EPS 2	Case EPS 3
Sex/age at diagnosis (years)	M/19	F/64	F/39
Primitive nephropathy	CAKUT	Diabetes	MPGN
Status at EPS diagnosis	HD	KTx	KTx
Time from peritoneal dialysis arrest to EPS diagnosis (months)	5	18	55
Peritoneal dialysis modality	CAPD	CAPD	CAPD
Time on peritoneal dialysis (months)	21	90	164
Peritonitis episodes (*n*)	3	ND	8
Microorganisms	*S. aureus* (3)	ND	*E. coli* (3) *S. aureus* (3) *P. aeruginosa* (2)
Glucose overexposure	Yes	No	Yes
Chlorhexidine use	No	No	No
Ciclosporin	No	No	Yes
Everolimus	No	No	Yes

Clinical and radiological diagnostic criteria of EPS			
Clinical symptoms/hemoperitoneum	Yes/no	Yes/yes	Yes/yes
Abdominal CT findings	Intestinal subocclusion Adherence	Loculated ascites Peritoneal thickening and calcifications	Loculated severe ascites Peritoneal thickening and calcifications

Treatment of EPS			
Azathioprine (mg/day)	50	75	50
Tamoxifen (mg/day)	/	20	10
Dexamethasone (mg/day)	/	32	8
Current state	HD	Died (septicemia)	KTx
Follow-up (months)	237	ND	57
Outcome	Nausea Abdominal pain	ND	Recurrent ascites, bacterial peritonitis (2)

EPS: encapsulating peritoneal sclerosis; M: man; W: woman; CAKUT: congenital abnormality of kidney and urinary tract; MPGN: membranoproliferative glomerulonephritis; HD: haemodialysis; KTx: kidney transplantation CAPD: continuous ambulatory peritoneal dialysis; ND: no data available.

**Table 3:** Results of immunohistochemical quantifications of mesothelio-mesenchymal transdifferentiation process, evaluation of interstitial inflammation, and vasculature in peritoneal biopsies samples from controls, case of acute peritonitis, and patients with encapsulating peritoneal sclerosis.

	Control 1	Control 2	Case of acute peritonitis	Case EPS 1	Case EPS 2	Case EPS 3
Markers of mesothelial cells phenotype						
AE1/AE3						
Mesothelium	(+++)	(+++)	(+++)	0	(0)	0
Interstitium	0	0	0	(+++)	(+++)	(+++)
Calretinin						
Mesothelium	(+++)	(+++)	(+++)	0	0	0
Interstitium	0	0	(+/−)	(++)	(+)	(+)

Immunohistochemical phenotyping of inflammatory infiltrating cells						
CD4^*∗*^	19.6	11.5	8.85	1.05	10.8	ND
CD8^*∗*^	1.15	0	1.05	0.9	2.25	130
CD68^*∗*^	0.9	1.25	4.7	2.75	1.66	18.8
CD20^*∗*^	3	0	0	3	10	1

Markers of endothelial cells phenotype						
CD31^#^	0.95	0.8	4.2	6.5	0.5	ND

Markers of mesenchymal cells phenotype						
Vimentin						
Mesothelium	0	0	(+)	(+++)	(+++)	(+)
Interstitium	0	0	(+)	(+++)	(+++)	(+++)

Markers of myofibroblasts						
Alpha SMA	0	0	(+)	(+++)	(+++)	(+++)

EPS: encapsulating peritoneal sclerosis; SMA: alpha-smooth muscle actin. ND: no data available (insufficiency of biopsy material). Semiquantitative score analysis: expression; high: (+++), moderate: (++), low: (+), and absent: 0. Score of quantitative evaluations: (*∗*) mean number of cells or (#) vessels per field (details of all immunostaining analysis are described in Material and Methods).
